# Long-term Psychoactive Medications, Polypharmacy, and Risk of Suicide and Unintended Overdose Death Among Midlife and Older Women Veterans

**DOI:** 10.1007/s11606-022-07592-4

**Published:** 2022-08-30

**Authors:** Carolyn J. Gibson, Yixia Li, Guneet K. Jasuja, Salomeh Keyhani, Amy L. Byers

**Affiliations:** 1 San Francisco VA Health Care System, 4150 Clement Street, 116P, San Francisco, CA 94121 USA; 2 University of California, San Francisco, San Francisco, USA; 3 NCIRE-The Veterans Health Research Institute, San Francisco, USA; 4 Center for Healthcare Organization and Implementation Research (CHOIR), Edith Nourse Rogers Memorial VA Medical Center, Bedford, USA; 5 Boston University School of Medicine, Boston, USA

**Keywords:** suicide, mortality, prescriptions, Veterans, women’s health

## Abstract

**Background:**

Rates of suicide and unintended overdose death are high among midlife and older women, yet there is paucity of data identifying women at greatest risk. Psychoactive medications, commonly prescribed and co-prescribed in this population, may serve as salient indicators of risk for these outcomes.

**Objective:**

To determine whether long-term psychoactive medications and psychoactive polypharmacy predict risk of suicide and unintended overdose death among midlife and older women Veterans above and beyond other recognized factors.

**Design:**

Longitudinal cohort study

**Participants:**

Women Veterans aged ≥ 50 with at least one Veterans Health Administration (VHA) clinical encounter in FY2012–2013.

**Main Measures:**

Long-term psychoactive medications (opioids, benzodiazepines, sedative-hypnotics, antidepressants, antipsychotics, and antiepileptics, prescribed for ≥ 90/180 days) and psychoactive polypharmacy (overlapping for ≥ 1 day) from VHA pharmacy records; suicide and unintended overdose death through December 31, 2018.

**Key Results:**

In this national sample of 154,558 midlife and older women Veterans (mean age 63.4, SD 9.3 years), 130 died by suicide and 175 died from unintentional overdose over an average of 5.6 years. In fully adjusted models, long-term opioids (hazard ratio (HR) 2.01, 95% CI 1.21–3.35) and benzodiazepines (HR 2.99, 95% CI 1.82–4.91) were associated with death by suicide; opioids (HR 3.62, 95% CI 2.46–5.34), benzodiazepines (HR 2.77, 95% CI 1.73–4.42), sedative-hypnotics (HR 1.87, 95% CI 1.06–3.29), antidepressants (HR 1.47, 95% CI 1.03–2.12), antipsychotics (HR 1.81, 95% CI 1.02–3.22), and antiepileptics (HR 2.17, 95% CI 1.48–3.19) were associated with unintended overdose death. Women who were co-prescribed ≥ 3 psychoactive medications had over 2-fold increased risk of suicide (HR 2.83, 95% CI 1.65–4.84) and unintended overdose death (HR 2.60, 95% CI 1.72–3.94).

**Conclusions:**

Long-term psychoactive medications and psychoactive medication polypharmacy were important indicators of risk for death by suicide and death by unintended overdose among midlife and older women Veterans, even after accounting for psychiatric and substance use disorders.

**Supplementary Information:**

The online version contains supplementary material available at 10.1007/s11606-022-07592-4.

## INTRODUCTION

Among women, the highest rates of suicide occur in midlife.^1^ Psychoactive medications, including opioids, benzodiazepines, sedative-hypnotics, antidepressants, antipsychotics, and antiepileptics, are also commonly prescribed and co-prescribed among women during this period in the lifespan.^2,3^ Psychoactive medications are often used in self-poisoning,^4^ the most frequently used method for suicide attempts among women,^5^ and are common in individuals who attempt or die by suicide by other means.^6,7^

Overdose mortality, including both overdose-related suicide and unintended overdose death, has also increased by 350–500% among women aged 50 and older in the past two decades.^8^ Psychoactive medications and psychoactive polypharmacy, particularly opioids alone or co-prescribed with benzodiazepines, antidepressants,^9^ and/or antiepileptics such as gabapentin,^10^ are frequently implicated in overdose mortality and overdose-related emergency visits.^9,11–13^ These medications and related polypharmacy may contribute to suicide and overdose risk through multiple pathways not fully captured by other predictors. For example, common psychiatric diagnoses frequently underlying psychoactive medications are consistent predictors of suicide and overdose, particularly among women.^14,15^ Psychoactive medications may be indicators of underlying distress and disease severity, and/or provide access to lethal means. Polypharmacy may also be an important indicator of complex comorbidities that increase risk of suicidal behaviors.

Recognizing and understanding markers of suicide and overdose risk among women Veterans is a priority for the Veterans Health Administration (VHA). Psychoactive medication use is high in this population, with several studies identifying potentially unsafe prescribing patterns for these medications among women Veterans.^16–19^ In this study, we used national VHA data to examine associations between psychoactive medication use and mortality among midlife and older women Veterans. We hypothesized that long-term use of psychoactive medications and psychoactive polypharmacy would be strong markers of risk associated with both death by suicide and unintended death by overdose.

## METHODS

### Data Source

The cohort for this longitudinal study was drawn from the linkage of four national databases: (1) the VHA’s National Patient Care Database, which includes all inpatient and outpatient VHA services; (2) the VHA’s Pharmacy Managerial Cost Accounting National Data Extract, which includes prescribed medications, prescription dates, and number of days’ supply for each prescription; (3) Centers for Medicare and Medicaid Services (CMS) data, which includes medical claims/diagnoses; and (4) the VHA’s Mortality Data Repository (MDR), which includes cause-specific death information. The primary analytic cohort was comprised of Veterans aged 50 and older, identified as female in the VHA medical record and without a documented gender identity disorder, and with at least one VHA clinical encounter in fiscal years (FY) 2012–2013. In this primary analytic cohort, patients with at least one psychoactive medication prescription during the baseline period, including opioids, benzodiazepines, sedative-hypnotics, antidepressants, antipsychotics, and antiepileptics (“on psychoactive medications”) were matched 1:1 on age and index date to a comparison sample of patients without psychoactive medication prescriptions (“not on psychoactive medications”) during the same period. The index date for each Veteran on psychoactive medications was defined as the first prescription date for any psychoactive medication included in these analyses during the baseline period FY2012–2013. For Veterans not on psychoactive medications, the index date was the date of the first clinical encounter during the baseline period. The study was approved by the institutional review boards of the [institution blinded for peer review] and the Research and Development Committee of the study was approved by the institutional review boards of the University of California, San Francisco and the Research and Development Committee of the San Francisco VA Health Care System.

### Variables

#### Exposure: Long-term Psychoactive Medications and Psychoactive Polypharmacy

Prescribed psychoactive medications (opioids, benzodiazepines, sedative-hypnotics, antidepressants, antipsychotics, antiepileptics) were assessed by data abstraction from VHA pharmacy data for each Veteran during the baseline period (FY2012–2013). Long-term psychoactive medication was defined as medication prescribed for ≥ 90 out of 180 days,^20–22^ with at least the date of the first prescription occurring before the end of the baseline period (FY2013). Psychoactive polypharmacy was defined as co-prescribing of medications in different classes during the baseline period, with medications overlapping by ≥ 1 day. These were categorized as 0, 1, 2, and ≥ 3 prescriptions. This approach includes both the conventional definition of polypharmacy (i.e., concurrent long-term use of multiple medications)^23^ and patterns such as cross-tapering and/or switching medications or being prescribed a limited course of psychoactive medications for acute concerns. We have used this definition previously to examine polypharmacy with other medications.^21,24^

#### Outcomes: Death by Suicide and Death by Unintended Overdose

Death by suicide and death by unintended overdose were identified by MDR data (which includes cause and date of death), beginning from each Veteran’s index date (prescription date for those with psychoactive medication use during baseline; date of first clinical encounter at baseline for the comparison group) in FY2012–2013 through December 31, 2018. We defined death by suicide using International Classification of Diagnoses (ICD)-10 codes X60-X84, Y87.0; and death by unintended drug overdose using ICD-10 codes X40-X44.

#### Baseline Covariates

All covariates were selected a priori due to known or potential relationships with suicide and/or overdose risk.^15,25–27^ Age was defined as age at baseline, calculated from birth date documented in the medical record. Race was categorized as non-Hispanic White, non-Hispanic Black, Hispanic, and “other” (including race unknown), based on self-reported race in the medical record. Educational and income strata were classified by linking Veteran data to 2013 US Census data. Education was categorized according to college education completion in the Veteran’s zip code tabulation area (ZCTA) (≤ 25% vs. > 25% of the adult population); income was categorized by median ZCTA income tertiles, consistent with previously published methodology. Medical and psychiatric diagnoses were obtained from VHA and CMS medical records within the 2 years prior to and including the Veteran’s first clinical encounter or index prescription date during the baseline period. Medical diagnoses (hypertension, myocardial infarction, cardiovascular disease, diabetes mellitus, obesity, chronic pain, and sleep disorders), psychiatric diagnoses (depression, dysthymia, bipolar disorder, posttraumatic stress disorder, generalized anxiety disorder, panic disorder, specific phobia), and substance use disorders (alcohol use disorder, drug use disorder) were defined by ICD-9-CM codes.

### Statistical Analyses

Descriptive statistics were used to summarize demographic variables and clinical characteristics at baseline, overall and stratified by mortality outcomes. Fine-Gray proportional hazards models were used to examine risk of death by suicide and death by unintended overdose for women Veterans prescribed (1) long-term psychoactive medication and (2) psychoactive medication polypharmacy, accounting for competing risk of other deaths with follow-up time as the timescale (i.e., time to outcome from the index date) and follow-up censored at December 31, 2018. We generated three models for each outcome: (1) unadjusted, (2) model 1: adjusted for demographic variables and individual medical diagnoses, and (3) model 2: adjusted for demographic variables, medical diagnoses, and psychiatric comorbidity (including individual psychiatric diagnoses and individual substance use disorders). In exploratory analyses of potential differences in risk for midlife vs. older adults, interactions by age (50–64 and ≥ 65) were also tested. In secondary analyses, equivalent models with psychoactive polypharmacy as the independent variable were examined in a subsample that excluded women who were prescribed opioids during baseline. We conducted these secondary analyses to assess the influence of non-opioid psychoactive medications as predictors independent from opioids. Proportional hazards assumptions were evaluated graphically and statistically and determined to be satisfied for all models. Statistical tests for models were two-tailed, with significance set at *p* < 0.05. All analyses were performed using SAS version 9.4 (SAS Institute Inc, Cary, NC) and STATA version 16.1 (StataCorp, College Station, TX).

## RESULTS

### Characteristics of the Sample

The final analytic sample was comprised of 154,558 midlife and older women Veterans (mean age 63.4, standard deviation (SD) 9.3), followed for a mean of 5.6 (SD 2.0) years. The majority of the sample was non-Hispanic White (63%), and medical (64%) and psychiatric (38%) comorbidities were common. From the index date through December 31, 2018, death by suicide was documented in 130 women (mean follow-up duration 3.0 years, SD 1.9), and death by unintended drug overdose was documented in 175 women (mean follow-up duration 2.7 years, SD 2.0). Antidepressants were the most commonly prescribed long-term psychoactive medication (24%), and 10% of women were co-prescribed ≥ 3 psychoactive medications (Table 1). Despite increased risks for psychoactive medication use and psychoactive polypharmacy in older women,^28^ prescribing rates for all classes of medication and co-prescribing patterns were similar in women 50–64 and ≥ 65 (data not shown).

**Table 1:** Characteristics of the Sample at Baseline, Stratified by Mortality Outcomes*

	**Total** **(*n*** **= 154,558)**	**Death by suicide** **(*n*** **= 130)**	**Death by unintended overdose** **(*n*** **= 175)**
**Age, mean (SD)**	63.39 (9.33)	60.48 (6.69)	57.10 (5.40)
**Age,** ***n*** **(%)**			
50–64	108,712 (70.34)	106 (81.54)	166 (94.86)
≥ 65	45,846 (29.66)	24 (18.46)	…
**Race,** ***n*** **(%)**			
Non-Hispanic White	97,248 (62.92)	105 (80.77)	126 (72.00)
Non-Hispanic Black	25,329 (16.39)	…	29 (16.57)
Hispanic	764 (0.49)	…	…
Other or unknown	31,217 (20.20)	16 (12.31)	19 (10.86)
**Education,**† ***n*** **(%)**	63,485 (42.38)	62 (48.82)	70 (41.18)
**Income,** ***n*** **(%)**			
Low tertile (< $41,721)	48,885 (33.01)	46 (36.51)	60 (35.29)
Middle tertile	49,020 (33.10)	43 (34.13)	45 (26.47)
High tertile (> $54,784)	50,187 (33.89)	37 (29.37)	65 (38.24)
**Medical diagnoses**‡			
Any medical diagnosis	99,171 (64.16)	88 (67.69)	134 (76.57)
Hypertension	80,044 (51.79)	63 (48.46)	105 (60.00)
Myocardial infarction	3,736 (2.42)	…	…
Cardiovascular disease	11,886 (7.69)	11 (8.46)	23 (13.14)
Diabetes mellitus	32,221 (20.85)	31 (23.85)	33 (18.86)
Obesity	34,907 (22.59)	26 (20.00)	42 (24.00)
Chronic pain	10,717 (6.93)	17 (13.08))	50 (28.57)
Sleep disorder	25,517 (16.51)	26 (20.00)	47 (26.86)
**Psychiatric diagnoses**‡			
Any psychiatric disorder	58,327 (37.74)	94 (72.31)	141 (80.57)
Depression§	48,741 (31.54)	71 (54.62)	121 (69.14)
Dysthymia	10,154 (6.57)	19 (14.62)	24 (13.71)
Bipolar disorder	7,928 (5.13)	36 (27.69)	42 (24.00)
Posttraumatic stress disorder	15,329 (9.92)	32 (24.62)	52 (29.71)
Anxiety disorders	8,787 (5.69)	19 (14.62)	35 (20.00)
Generalized anxiety disorder	5,497 (3.56)	12 (9.23)	14 (8.00)
Panic disorder	2,884 (1.87)	…	…
Specific phobia	1,632 (1.06)	…	16 (9.14)
Any substance use disorder	9,385 (6.07)	30 (23.08)	78 (44.57)
Alcohol use disorder	6,427 (4.16)	21 (16.15)	43 (24.57)
Drug use disorder	5,291 (3.42)	21 (16.15)	69 (39.43)
**Long-term psychoactive medication use (**≥ **90 days)**‖			
Opioids	12,249 (7.93)	22 (16.92)	46 (26.29)
Benzodiazepines	7,577 (4.90)	24 (18.46)	26 (14.86)
Sedative-hypnotics	5,764 (3.73)	12 (9.23)	17 (9.71)
Antidepressants	37,737 (24.42)	50 (38.46)	71 (40.57)
Antipsychotics	5,690 (3.68)	17 (13.08)	22 (12.57)
Antiepileptics	18,342 (11.87)	26 (20.00)	48 (27.43)
**Psychoactive polypharmacy** **(**≥ **1 day overlap)** ¶**,**#			
0	77,279 (50.00)	43 (33.08)	66 (37.71)
1	40,340 (26.10)	23 (17.69)	16 (9.14)
2	21,350 (13.81)	24 (18.46)	30 (17.14)
≥ 3	15,589 (10.09)	40 (30.77)	63 (36.00)

Note: Ellipses denote that individual cell count was too small to report based on data use agreement; these values were included in any count

*Mortality outcomes from index date through 12/31/2018

†Live in area where > 25% of population has attended college

‡Diagnoses at index date or within 2 years prior to index date

§Depression includes major depressive disorder, depression NOS

‖Long-term use defined as from index prescription date during baseline and 180 days (6 months) forward with ≥ 90 of 180 days of medication use

¶All classes (opioids, benzodiazepines, sedative-hypnotics, antidepressants, antipsychotics, antiepileptics)

#Concurrent use of medications in different classes, with medication use overlapping by ≥ 1 day

### Long-term Psychoactive Medication Use and Death by Suicide and Unintended Overdose Death

All classes of psychoactive medications were 1.5–3 times more common among women who died by suicide or unintended overdose than the proportions seen for the overall sample (e.g., 5% of women in the total sample, and 18% of women who died by suicide, were prescribed long-term benzodiazepines; 8% of women in the total sample, and 26% of women who died by unintended overdose, were prescribed long-term opioids; Table 1).

In unadjusted Fine-Gray analyses, women prescribed any class of long-term psychoactive medications at baseline had a 2- to over 4-fold increased risk of dying by suicide (Table 2), and a 2- to over 5-fold increased risk of death by unintended overdose (Table 3), than those not prescribed these medications. For both outcomes, all associations were attenuated but still significant with adjustment for demographic variables and medical comorbidities in model 1. With further adjustment for psychiatric and substance use disorder comorbidities (model 2), only long-term opioids and benzodiazepines remained significantly associated with death by suicide (Table 2). In contrast, long-term opioids, benzodiazepines, non-benzodiazepine sedative-hypnotics, antidepressants, antipsychotics, and antiepileptics remained significantly associated with death by unintended overdose in fully adjusted models (Table 3). In exploratory analyses, a significant interaction by age was observed for the relationship between antidepressants and unintentional overdose death, though power was limited to examine this further due to the small number of women > 65 in the sample who died by unintended overdose (data not shown).

**Table 2:** Associations Between Long-term Psychoactive Medication Use, Psychoactive Polypharmacy, and Death by Suicide

	**Unadjusted** **HR (95%CI)**	**Model 1** **HR (95% CI)**	**Model 2** **HR (95% CI)**
Long-term psychoactive medications			
Opioids	2.34 (1.43–3.81)†	2.21 (1.33–3.67)†	2.01 (1.21–3.35)†
Benzodiazepines	4.25 (2.65–6.82)‡	3.77 (2.31–6.15)‡	2.99 (1.82–4.91)‡
Sedative-hypnotics	2.62 (1.41–4.87)†	2.44 (1.32–4.52)†	1.84 (0.97–3.47)
Antidepressants	2.06 (1.43–2.95)‡	1.94 (1.32–2.84)†	1.50 (0.99–2.28)
Antipsychotics	4.12 (2.44–2.98)‡	4.02 (2.36–6.86)‡	1.84 (0.98–3.44)
Antiepileptics	2.14 (1.38–3.32)†	1.97 (1.24–3.13)†	1.37 (0.85–2.21)
Psychoactive polypharmacy			
1	1.13 (0.68–1.90)	1.21 (0.71–2.05)	1.28 (0.76–2.16)
2	1.81 (1.05–3.11)*	1.78 (0.99–3.21)	1.49 (0.82–2.72)
3+	4.63 (2.96–7.25)‡	4.62 (2.82–7.57)‡	2.83 (1.65–4.84)‡

^a^Model 1: Adjusted for demographics (race, education, income), medical disorder (hypertension, myocardial infarction, cardiovascular disease, diabetes mellitus, obesity, chronic pain, sleep disorders)

^b^Model 2: Adjusted for demographics (race, education, income), medical disorder (hypertension, myocardial infarction, cardiovascular disease, diabetes mellitus, obesity, chronic pain, sleep disorders), psychiatric disorder (depression, dysthymia, bipolar disorder, posttraumatic stress disorder, generalized anxiety disorder, panic disorder, or specific phobia), and substance use disorder (alcohol use disorder, drug use disorder)

*p < .05, †*p* < .01, ‡*p* < .001

**Table 3:** Associations Between Long-term Psychoactive Medication Use, Psychoactive Polypharmacy, and Death by Unintended Overdose

	**Unadjusted** **HR (95%CI)**	**Model 1** **HR (95% CI)**	**Model 2** **HR (95% CI)**
Long-term psychoactive medications			
Opioids	5.47 (3.86–7.75)‡	3.83 (2.61–5.63)‡	3.62 (2.46–5.34)‡
Benzodiazepines	4.39 (2.85–6.76)‡	3.22 (2.05–5.07)‡	2.77 (1.73–4.42)‡
Sedative-hypnotics	2.90 (1.70–4.95)‡	2.24 (1.28–3.94)†	1.87 (1.06–3.29)*
Antidepressants	2.12 (1.53–2.94)‡	1.75 (1.25–2.46)†	1.47 (1.03–2.12)*
Antipsychotics	3.51 (2.12–5.82)‡	2.88 (1.69–4.89)‡	1.81 (1.02–3.22)*
Antiepileptics	3.39 (2.39–4.80)‡	2.53 (1.74–3.67)‡	2.17 (1.48–3.19)‡
Psychoactive polypharmacy			
1	0.55 (0.31–0.97)*	0.49 (0.28–0.89)*	0.57 (0.31–1.03)
2	1.80 (1.14–2.86)*	1.48 (0.92–2.37)	1.40 (0.86–2.30)
3+	4.97 (3.44–7.17)‡	3.39 (2.28–5.04)‡	2.60 (1.72–3.94)‡

^a^Model 1: Adjusted for demographics (race, education, income), medical disorder (hypertension, myocardial infarction, cardiovascular disease, diabetes mellitus, obesity, chronic pain, sleep disorders)

^b^Model 2: Adjusted for demographics (race, education, income), medical disorder (hypertension, myocardial infarction, cardiovascular disease, diabetes mellitus, obesity, chronic pain, sleep disorders), psychiatric disorder (depression, dysthymia, bipolar disorder, posttraumatic stress disorder, generalized anxiety disorder, panic disorder, or specific phobia), and substance use disorder (alcohol use disorder, drug use disorder)

*p < .05, †*p* < .01, ‡*p* < .001

### Psychoactive Polypharmacy and Death by Suicide and Unintended Overdose Death

**Figure 1:**
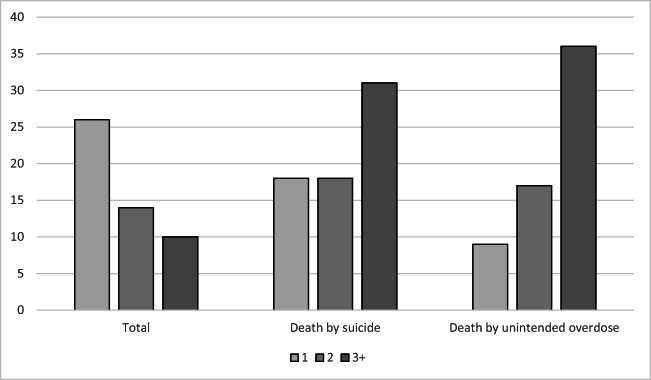
Proportion of women prescribed psychoactive medications in total sample and among those who died by suicide and unintended overdose.

Compared to the overall sample, psychoactive polypharmacy was more than 3 times as common among women who died by suicide (31%) and unintended overdose (36%; Table 1, Fig. 1). In unadjusted Fine-Gray analyses, women prescribed ≥ 3 psychoactive medications during baseline had over 4-fold increased risk of death by suicide (Table 2) and nearly 5-fold increased risk of death by unintended overdose compared to those not prescribed psychoactive medications (Table 3). For both outcomes, these associations were attenuated but still significant in models further adjusted by demographic variables and medical comorbidities as well as psychiatric and substance use disorder comorbidities (Table 2, Table 3). In fully adjusted models run in the subsample excluding data from women prescribed opioids (*n* = 86,166), results were attenuated for suicide (HR 2.10, 95% CI 0.95–4.61) and largely equivalent for unintended overdose death (HR 2.76, 95% CI 1.36–5.63). In exploratory analyses, no significant interactions for polypharmacy and either mortality outcome by age were observed (data not shown).

## DISCUSSION

In this national sample of midlife and older women Veterans enrolled in VHA care, we examined associations between long-term psychoactive medication use, psychoactive polypharmacy, and risk of death by suicide and unintended overdose. Independent of known risk factors, long-term opioids and benzodiazepines were associated with a statistically significant 2-fold and higher risk of death by suicide, and long-term opioids, benzodiazepines, non-benzodiazepine sedative-hypnotics, antidepressants, antipsychotics, and antiepileptics were associated with 1.5- to over 3-fold higher risk of unintended overdose death. Women who were prescribed three or more psychoactive medications had an almost 3-fold higher risk of death by both suicide and unintended overdose. These findings highlight long-term and co-prescribed psychoactive medications as important indicators of increased risk for suicide and unintended overdose death among midlife and older women.

Psychoactive medications are regularly prescribed for depression, anxiety, posttraumatic stress disorder, insomnia, and chronic pain, common conditions among midlife and older women that are also primary predictors of suicide and overdose mortality.^15,29^ The results of this study suggest that these diagnoses partially explained the relationship between psychoactive medications and outcomes of interest. However, long-term opioids and benzodiazepines remained an important prognostic factor for death by suicide, and long-term opioids, benzodiazepines, sedative-hypnotics, antidepressants, antipsychotics, and antiepileptics remained an important prognostic factor for death by unintended overdose, above and beyond these diagnoses. Long-term use of these medications may therefore signal the underlying severity of psychiatric comorbidity and/or additional unmeasured factors^30^ contributing to suicide and overdose mortality.

Consistent with the current study, both opioids and benzodiazepines have been consistently associated with both suicide-related and unintended overdose mortality in studies of general populations,^31,32,8,33,12^ with elevated risk related to long-term use.^34^ Additionally, while all examined long-term psychoactive medications were associated with unintended overdose death, one of the strongest associations was seen with long-term antiepileptics. This finding may relate to a growing body of evidence highlighting risks for misuse and overdose related to gabapentinoids.^35,36^ There has been exponential growth in the use of these widely prescribed antiepileptics over the past two decades,^37^ particularly among midlife and older women for common conditions including pain^38^ mood symptoms, sleep difficulty,^39^ and menopausal hot flashes and night sweats.^40,41^

We also found a pronounced risk for death by suicide and unintended overdose death among women co-prescribed multiple psychoactive medications. Previous studies have shown that psychoactive polypharmacy is common among specific Veteran populations, including midlife women Veterans with chronic pain^21^ and Veterans with PTSD,^17^ and it has been associated with increased risk for overdose and suicide-related behavior in younger Veterans.^11^ Polypharmacy compounds overdose risks related to central nervous system and respiratory depression, as well as functional and cognitive impairment that may influence suicidal behaviors.^34,35,42^ Additionally, psychoactive polypharmacy reflects complex comorbidity, and may indicate underlying symptom severity.^11^ Most findings linking psychoactive polypharmacy to suicide-related or unintended overdose have involved opioids in addition to benzodiazepines and/or other psychoactive medications.^10,43,44^ Our findings suggest that psychoactive polypharmacy may be important in and of itself, as women prescribed three or more psychoactive medications had equivalently elevated risk for unintended overdose death regardless of whether opioids were included in the categorization.

These findings have important clinical implications for midlife and older women and their health care providers. By identifying women with long-term and co-prescribed psychoactive medications, prevention efforts can be strategically implemented, such as appropriate monitoring of those who start on certain medications and referral of patients to specialty mental health care. Risk for suicide and unintended overdose may be mitigated with pragmatic steps including limiting the number of pills dispensed with each prescription, routine counseling around lethal means restriction, and promoting safe disposal of old and excess medications. Of note, we cannot determine in this study whether or to what extent any of these preventive strategies were implemented. These findings also highlight the importance of the availability of safe and effective evidence-based psychotherapies for psychiatric and related conditions, which may reduce reliance on long-term psychoactive medications while potentially alleviating multiple comorbidities.^45–47^ This is particularly relevant for examined medications with known risks and limited benefits related to long-term use, such as benzodiazepines^42^ and sedative-hypnotics.^16^ Overall, long-term and co-prescribed psychoactive medications may represent an important indicator of suicide and overdose risk during this vulnerable period. In addition to potential lethality, they may also capture complex comorbidity and a range of often co-occurring, frequently unmeasured risks.

Several limitations should be considered in interpreting these findings. Longitudinal trends including dose of prescribed medications, psychoactive medications prescribed after the baseline period, and changes in comorbidities over time were not examined and may influence suicide and overdose risk. We relied on ICD codes for psychiatric diagnoses and substance use disorders. We cannot determine aspects of these diagnoses that may be important for suicide and/or overdose risk, including symptom severity and acuity and whether documented diagnoses were chronic, unremitting, or in remission. We cannot determine with these data if observed death by suicide or unintended overdose death were directly or causally linked to the prescribed psychoactive medications examined. Further, we cannot determine if any unintended overdose deaths represented misclassifications of death by suicide. Pharmacy data was limited to VHA records, and we cannot account for non-VHA prescriptions. We used a definition of polypharmacy that may encompass multiple prescribing practices with differing risks and implications, including prescription transitions and cross-tapering as well as long-term ongoing polypharmacy. This study uses data from midlife and older women Veterans who use VHA health care, a population at elevated risk for suicide and overdose with high rates of medical and mental health comorbidities.^49^ Results may not be generalizable to women Veterans who do not use VHA health care, or to women in the general population.

Despite these limitations, this study has multiple strengths. This study is one of few to examine suicide and overdose death in midlife and older women, a population with increasing suicide rates^48^ and overdose mortality.^12^ We examined a large, diverse, nationally representative sample of midlife and older women Veterans who utilize VHA care, and accounted for a wide range of demographic and clinical factors to assess for independent relationships and limit confounding. Despite inherent limitations, the use of real-world data allows for prescribing patterns and suicide and overdose mortality as documented and managed within a large, integrated health care system. The current findings identify specific long-term and co-prescribed psychoactive medications as an indicator for death by suicide and unintentional overdose death within a high-risk period in the lifespan, which may help to inform prevention efforts in the VHA and other health care settings.

Rates of death by suicide and unintended overdose have risen dramatically among midlife and older women, but little is known about potential and preventable drivers of increased risk during this vulnerable period in the lifespan. Our findings from national VHA data suggest that among midlife and older women, long-term opioids, benzodiazepines, and psychoactive polypharmacy are important indicators of suicide risk, and long-term opioids, benzodiazepines, sedative-hypnotics, antidepressants, antipsychotics, antiepileptics, and psychoactive polypharmacy are important indicators of overdose mortality risk. These medications are commonly prescribed and co-prescribed among midlife and older women, highlighting the importance of monitoring, routine screening and mitigation for suicide and overdose risks, and deprescribing as appropriate for safe and effective comprehensive care for this vulnerable population.

## Supplementary Information

ESM 1 (DOCX 17 kb)
